# Validation of a redesigned pan-poliovirus assay and real-time PCR platforms for the global poliovirus laboratory network

**DOI:** 10.1371/journal.pone.0255795

**Published:** 2021-08-06

**Authors:** Hong Sun, Chelsea Harrington, Nancy Gerloff, Mark Mandelbaum, Stacey Jeffries-Miles, Lea Necitas G. Apostol, Ma. Anne-Lesley D. Valencia, Shahzad Shaukat, Mehar Angez, Deepa K. Sharma, Uma P. Nalavade, Shailesh D. Pawar, Elisabeth Pukuta Simbu, Seta Andriamamonjy, Richter Razafindratsimandresy, Everardo Vega

**Affiliations:** 1 Division of Viral Diseases, National Center for Immunization and Respiratory Diseases, Centers for Disease Control and Prevention, Atlanta, Georgia, United States of America; 2 Research Institute for Tropical Medicine, Muntinlupa City, Philippines; 3 National Institute of Health, Islamabad, Pakistan; 4 National Institute of Virology, Mumbai, India; 5 National Institute of Biomedical Research, Kinshasa, Democratic Republic of the Congo, Congo; 6 Virology Unit, Institut Pasteur de Madagascar, Antananarivo, Madagascar; Hong Kong Children’s Hospital, HONG KONG

## Abstract

Surveillance and detection of polioviruses (PV) remain crucial to monitoring eradication progress. Intratypic differentiation (ITD) using the real-time RT-PCR kit is key to the surveillance workflow, where viruses are screened after cell culture isolation before a subset are verified by sequencing. The ITD kit is a series of real-time RT-PCR assays that screens cytopathic effect (CPE)-positive cell cultures using the standard WHO method for virus isolation. Because ITD screening is a critical procedure in the poliovirus identification workflow, validation of performance of real-time PCR platforms is a core requirement for the detection of poliovirus using the ITD kit. In addition, the continual update and improvement of the ITD assays to simplify interpretation in all platforms is necessary to ensure that all real-time machines are capable of detecting positive real-time signals. Four platforms (ABI7500 real-time systems, Bio-Rad CFX96, Stratagene MX3000P, and the Qiagen Rotor-Gene Q) were validated with the ITD kit and a redesigned poliovirus probe. The poliovirus probe in the real-time RT-PCR pan-poliovirus (PanPV) assay was re-designed with a double-quencher (Zen^™^) to reduce background fluorescence and potential false negatives. The updated PanPV probe was evaluated with a panel consisting of 184 polioviruses and non-polio enteroviruses. To further validate the updated PanPV probe, the new assay was pilot tested in five Global Polio Laboratory Network (GPLN) laboratories (Madagascar, India, Philippines, Pakistan, and Democratic Republic of Congo). The updated PanPV probe performance was shown to reduce background fluorescence and decrease the number of false positives compared to the standard PanPV probe.

## Introduction

Poliovirus surveillance is essential to the success of the Global Poliovirus Eradication Initiative (GPEI) [1, 2]. With poliovirus eradication nearing, rapid detection of polioviruses from specimens collected through acute flaccid paralysis (AFP) and environmental surveillance systems is crucial to monitor eradication progress. Improving methods and procedures by increasing sensitivity and robustness is a major objective of the Global Polio Laboratory Network (GPLN). Molecular methods, like real-time reverse transcription PCR (rRT-PCR), can identify and distinguish wild and vaccine-like polioviruses isolated from AFP cases or environmental sources [3–6], but algorithms rely on sequencing as the gold standard to provide final verification. Intratypic differentiation (ITD) by rRT-PCR is key to the GPLN surveillance workflow, to rapidly screen poliovirus isolates of programmatic importance after cell culture isolation and before verification of a subset of isolates by sequencing. Over the years, as technology and polio eradication needs have evolved, ITD assays have changed multiple times, from version 1 to version 6, to better meet the needs of the global network [7–9].

The rRT-PCR screening kit, ITD 5.0, consists of six assays (EV+Sabin quadruplex, PanPolio [PanPV] assay, wild poliovirus type 1 (WPV1), PV type 2 (PV2) assay, wild poliovirus type 3 (WPV3)-I and WPV3-II assay used in conjunction with a decision algorithm to identify polioviruses of programmatic importance to be referred for sequencing. Poliovirus sequences inform the molecular epidemiology of the virus to help guide vaccination campaigns. The ITD 5.0 suite of assays has been adapted and modified from the previous version, ITD 4.0; evaluations with the ABI7500 rRT-PCR system report 97.7%–99.1% specificity and 92%–100% sensitivity [7]. Most of the 114 accredited ITD laboratories use ABI7500 real-time PCR systems, but other instrument platforms, such as the Bio-Rad CFX96, Stratagene MX3000P, and Qiagen Rotor-Gene Q systems are used as well. The ability of the additional instruments to work with the poliovirus suite of diagnostic assays (e.g., with high sensitivity and specificity) is important to provide adequate global coverage in testing poliovirus.

Most real-time platforms, like ABI7500, Bio-Rad CFX96, and Stratagene MX3000P, use Peltier elements for the regulation of heating and cooling of samples, and can produce different results from the Rotor-Gene Q system, which uses a rotary mechanism in which samples are spun continuously and heated and cooled with air [10]. Differences between the two platform types are most apparent with assays using highly degenerate primers and probes, like the PanPV assay, which utilizes 21 mixed-bases and 8 deoxyinosine residues in order to identify all polioviruses [8, 9].

In this study, the PanPV assay was investigated because it showed an increased background signal on the Rotor-Gene Q platform that may lead to false-negative results. Accordingly, a new poliovirus probe was needed to reduce fluorescence background and improve sensitivity. Here, we report on an evaluation of ITD performance on different real-time PCR platforms and on pilot results for the novel poliovirus probe for the updated ITD 5.1 kit tested in five GPLN laboratories (Philippines, Pakistan, Madagascar, India and Democratic Republic of the Congo).

## Material and methods

### Virus isolates and RNA transcripts for real-time RT-PCR platform validations

A virus panel (n = 184) encompassing all PV serotypes (N = 158), non-polio enterovirus (NPEV) (N = 15), and non-enterovirus (NEV) (N = 11) (CPE-positive cultures but enterovirus-negative by ITD), was used to measure assay performance with the six ITD assays according to previously described methods (Table 1) [7]. Isolates were derived from stools of AFP surveillance cases collected between 1999–2015 from GPLN and sent to the Polio laboratory at the Centers for Disease Control and Prevention (CDC) for confirmation or processing.

**Table 1:** Summary of poliovirus, non-poliovirus and non-enterovirus isolates by serotype and genotype tested at the polio laboratory at CDC, Atlanta.

Serotype ^a^	Classification ^b^	No. of specimens (*n* = 184)
PV1	Sabin	10
	VDPV	11
	WEAF-B1	15
	SOAS	22
PV2	Sabin	15
	VDPV	16
	Wild	10
PV3	Sabin	12
	VDPV	14
	WPV AFR	13
	WPV SOAS	20
Non-polioviruses and Negative by Cell Culture Controls	NPEV^c^	15
	Negative^d^	11

^a^PV: poliovirus with number identifying serotype 1, 2, or 3.

^b^VDPV, (vaccine-derived poliovirus); West Africa-B1 genotype (WEAF-B1); South Asia genotype (SOAS); Wild poliovirus 3 isolates from African region, Wild poliovirus from South Asia region (WPV SOAS). Wild poliovirus 2 isolates were received from Asia, the Middle East, Africa, and the USA between 1999 and 2009; Salk Inactivated poliovirus (IPV) strains serotype 1 (Mahoney) and serotype 3 (Saukett) were positive in both EV and PanPV assays. The Mahoney strain was positive in the Sabin 1 assay and Saukett was positive in the Sabin 3 assay, both Sabin 1 and Sabin 3 were derived from Mahoney and Saukett, respectively.

^c^NPEV, non-polio enterovirus (coxsackievirus A4 [CV-A4], CV-A9, CV-A21, coxsackievirus B4 [CV-B4], CV-B5, echovirus 6 [E-6], E-9, E-11, E-12, EV-A71, EV-D68, Human Rhinovirus 47)

^d^Virus isolates with CPE but negative in ITD screening assays (non-enterovirus).

In addition, two plaque purified WPV1 isolates (Accession no. KY941931 and KY941934) from Human Rhabdomyosarcoma cell lines (RD cells, ATCC cat# CCL-136) were included; these strains last circulated in the African (AFR) and Eastern Mediterranean (EMR) WHO regions, respectively. A reference polio type 1 virus from the National Institute for Biological Standards and Control (NIBSC, UK) (Sabin 1, Accession no. AY184219) was used for the evaluation of the updated PanPV probe [11, 12].

Synthetic poliovirus RNA transcripts were used to assess the performance of the initially designed PanPV probes following a previously published method [7]. We generated three RNA transcripts derived from capsid viral protein 1 (VP1) sequences of the Sabin 1, Sabin 2, and Sabin 3 vaccine strains. All RNA standards were stored in single-use aliquots at −80°C until needed. Each ITD PCR reaction consisted of 10 μl of qScript^™^ XLT One-Step RT-qPCR ToughMix^®^ (Quanta Biosciences, Beverly, MA), 1 μl of primers/probe(s) mix (contained in the ITD kit; Centers for Disease Control and Prevention [CDC], Atlanta, GA), 8 μl RNase-free water, and 1 μl of template (virus culture supernatant or RNA).

### Real-time PCR platform evaluation

Each of the six ITD assays (EV+Sabin, PanPV, WPV1, PV type 2, WPV3-I and WPV3-II assays) was tested on four real-time PCR platforms: Applied Biosystems 7500 Real Time PCR System (ABI7500, ThermoFisher Scientific, Waltham, MA); Bio-Rad CFX96 (Bio-Rad Laboratories, Hercules, CA); Stratagene MX3000P (Agilent Technologies, Santa Clara, CA), and Rotor-Gene Q (Qiagen, Hilden, Germany). The ABI7500 is the most frequently used real-time PCR platform in the GPLN (90%) and is considered the “gold standard”. In order to accommodate the deoxyinosine-containing primers and probes, in earlier versions of the ITD run method, the ramp speed between annealing and extension for the ABI7500 was reduced to achieve higher specificity and sensitivity for the PanPV assay. The reduced ramp rate is a standard run method for the GPLN procedure [7]. The ramp speed for the Rotor-Gene would also require a slowdown but because the Rotor-Gene Q software does not have that option, an additional temperature step was added between annealing and extension. The thermocycling conditions for each PCR cycler are listed in Table 2.

**Table 2:** Validated ITD 5.0 run conditions on different real time systems.

ABI7500 & 7500 fast			CFX96 & MX3000P			Rotor-Gene Q		
PCR Cycles	95°C	15 sec	PCR Cycles	95°C	15 sec	PCR Cycles	95°C	15 sec
(40X)	50°C	45 sec	(40X)	50°C	45 sec	(40X)	50°C	45 sec
	25% ramp rate						61°C	20 sec
	95°C	15 sec		95°C	15 sec		95°C	15 sec

### Validation of updated PanPV assay

The virus panel consisting of 184 polioviruses and non-polio enteroviruses (Table 1) was tested with the current PanPV assay and with the best-performing updated PanPV probe on the Rotor-Gene Q at the Polio laboratory at CDC Atlanta. The same samples were then tested with the updated PanPV assay on the other PCR cyclers (ABI7500, CFX96, and MX3000P) using the run method described earlier (Table 2).

### Limit of Detection (LOD) for updated PV probe

The LOD and ITD reactions were run as previously described [7]. Briefly, WEAF-B1 WPV1, SOAS WPV1, and Sabin 1 isolates were tested in triplicate serial dilutions (10^7^ to 10^0^ CCID_50_·ml^-1^). The 95% LOD of the updated PanPV assay was determined by testing 20 replicates of the last dilution step with 95% positivity in the ABI7500. Thermocycling conditions were the same as described in Table 2.

### Pilot tests for updated PV probe in five GPLN laboratories

After completing the evaluation of the updated PV probe in the Polio and Picornavirus Laboratory at the CDC in Atlanta, newly developed Zen PV probes were combined with primers at the CDC. The PanPV Zen primer and probe mix was shipped to five GPLN laboratories for pilot testing of CPE positive virus isolates from AFP surveillance stools.

### Data management, statistical, and visual analysis

Any sample with cycle threshold (Ct)value < 40 was considered a positive a positive result for the assay-by-assay comparison. To analyze all real-time RT-PCR data, Ct values were recorded for each sample and target. Results were compiled and edited using R. The McNemar test was used for parallel testing analysis using the gmodels package in R [13]. Data visualizations were made using ggplot package in R and Prism 7.0, Graph Pad Software (San Diego, CA). To analyze background fluorescence between PanPV and updated PanPV from testing 32 WPV1 isolates, the raw fluorescence data from cycle 6, where background fluorescence stabilizes, was exported and compiled in Excel and R. The non-parametric Wilcoxon signed rank test was run in R to determine any significant difference in background fluorescence between the two assays.

### Ethical considerations

CDC’s internal program for Human Subjects Research Determination deemed that this study is categorized as public health non-research for the purpose of human subject regulations.

## Results

### High concordance between ABI7500, Rotor-Gene Q, CFX96, and MX3000P

**Fig 1:**
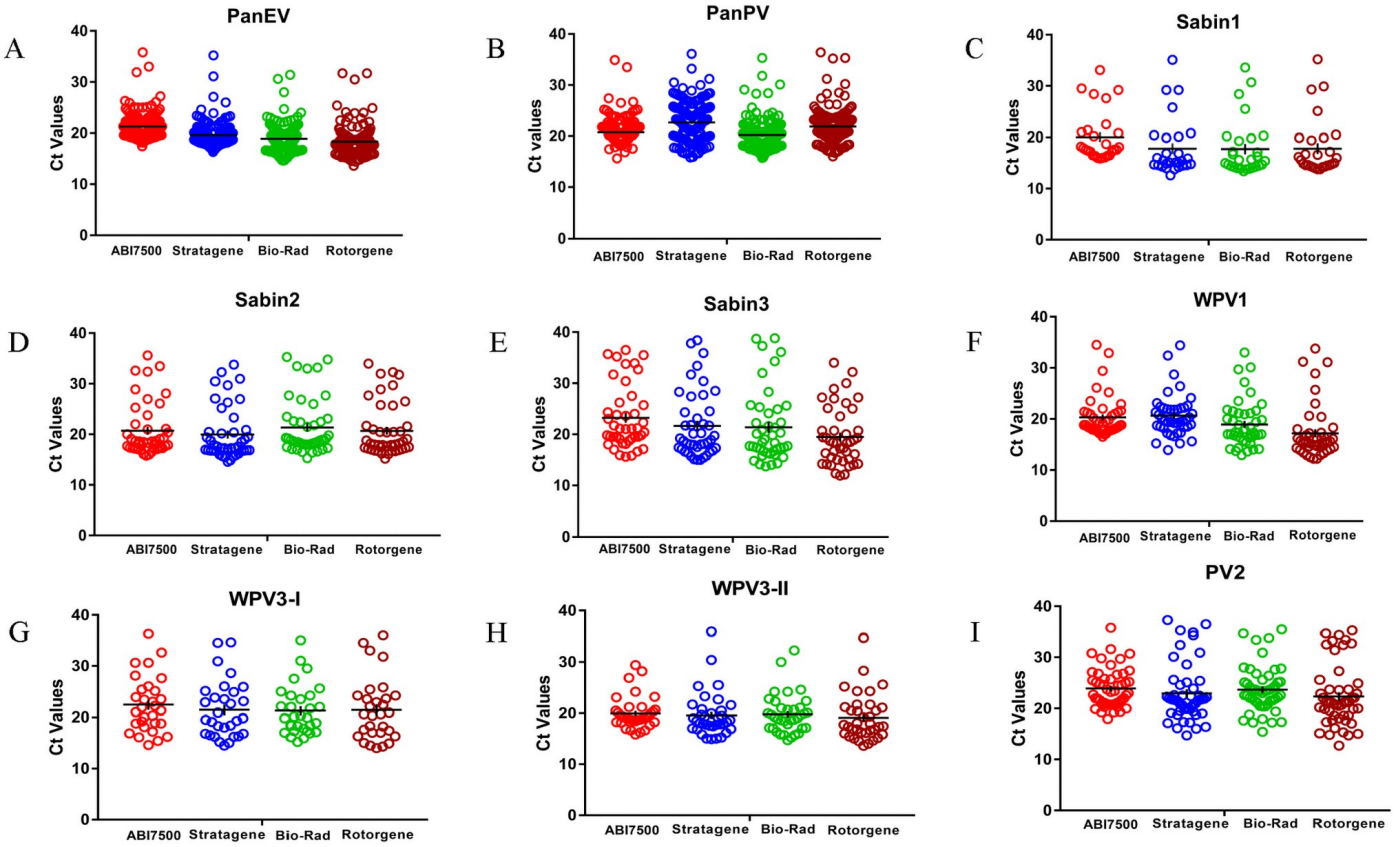
ITD 5.0 Assay results for stratagene (Mx3005P), ABI7500, bio-rad (CFX96) and Rotor-Gene Q (Qiagen). Assay performance against a standard virus panel (N = 184). (A) PanEV assay, 171 of 184 isolates; (B) Pan PV assay, 158 of 184 isolates; (C) Sabin 1 assay 28 of 184 isolates; (D) Sabin 2 assay 44 of 184 isolates; (E) Sabin 3 assay 43 of 184 isolates; (F) WPV1 assay 44 of 184 isolates; (G) WPV3-I assay 30 of 184 isolates; (H) WPV3-II assay 36 of 184 isolates; and (I) the PV Type 2 assay 50 of 184 isolates. The PanEV assay targets the 5’NTR, all other assays target the VP1 capsid region. The WPV3-I and WPV3-II assays target the WPV3 WEAF-B and SOAS variants, respectively.

A total of 158 poliovirus isolates from 1999–2015 were selected from the CDC database. All poliovirus serotypes (Table 1) were confirmed by VP1 sequence using standard methods [14]. Isolates were re-tested with the ITD 5.0 kit using ABI7500. All serotypes were detected by corresponding assays in the ITD (e.g., PanEV+, Sabin1+, PanPV+) including mixtures. The complete set of virus isolates was tested on the CFX96 and MX3000P, resulting in a 100% match for all six ITD assays (n = 184). Five of the ITD assays had 100% concordance between ABI7500 and Rotor-Gene Q; PanPV had 6 false-negatives out of 158 (3.8%) poliovirus isolates on Rotor-Gene Q. The six false negative virus isolates (clarified supernatant) were diluted 1:10 in Minimum Essential Media(MEM) and re-tested with the PanPV assay. All 6 virus isolates were positive after diluting and the results from all 6 assays were 100% concordant among the ABI7500, CFX96, MX3000P, and Rotor-Gene Q (Fig 1), indicating the false negatives were due to high background signals.

### Updated PV probe with Zen^™^ quencher was superior to the standard PanPV probe

**Fig 2:**
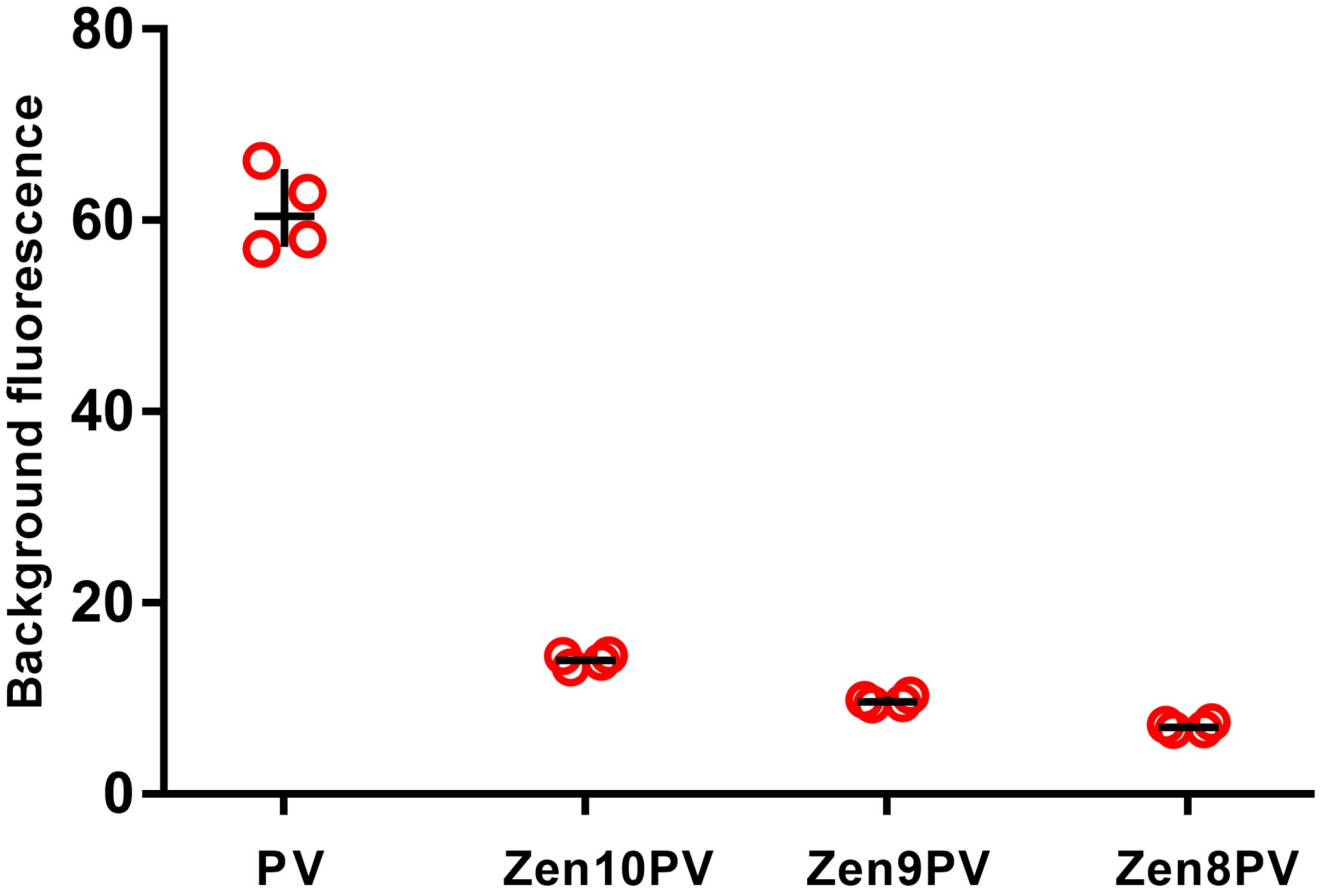
Comparison of the background fluorescence on the Rotor-Gene Q. Three updated PanPV probes with Zen^™^ quencher were tested along with the current PanPV probe to ascertain baseline background levels against synthetic control RNA. Fluorescence at cycle six was selected as representative of background levels for each assay.

**Fig 3:**
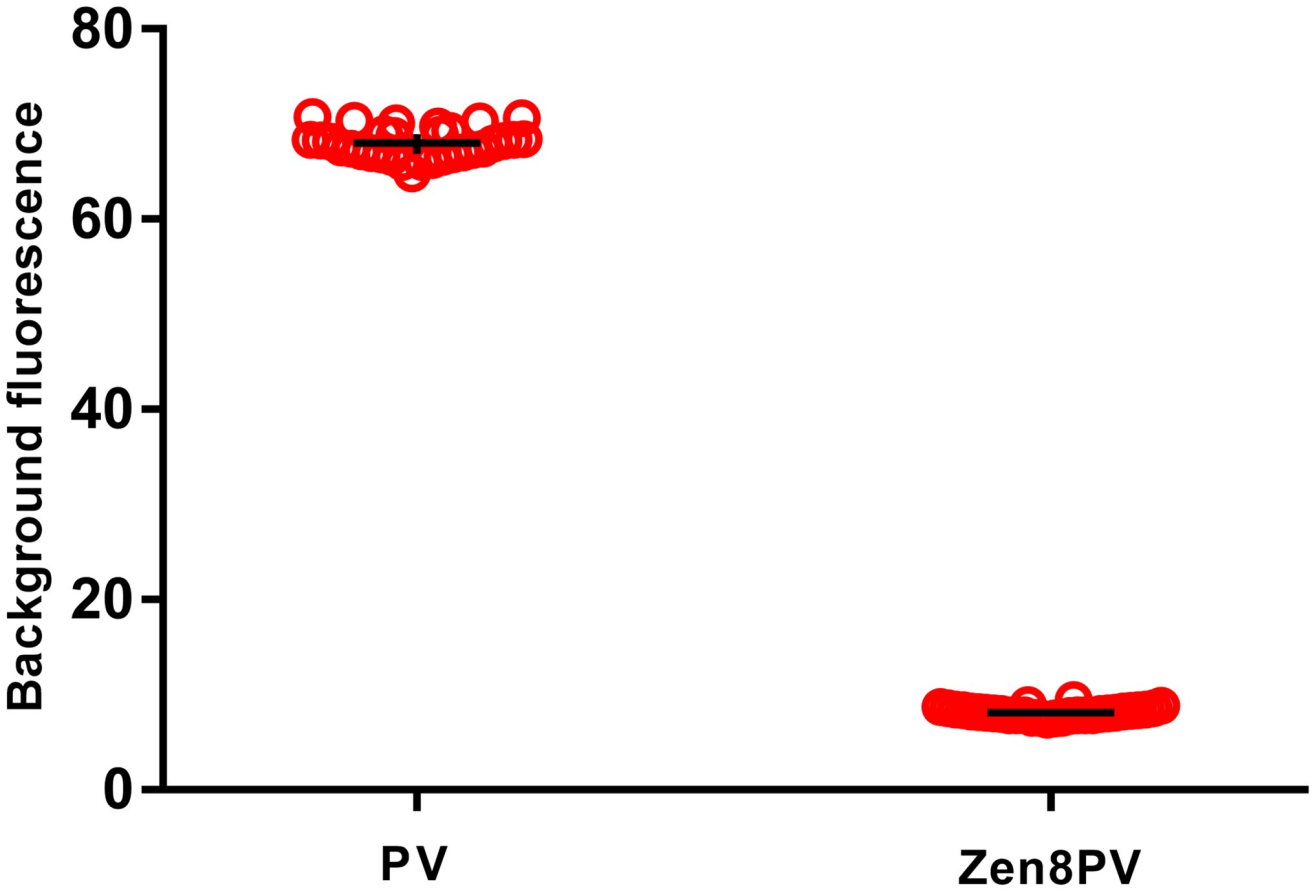
Comparison of the background fluorescence on the Rotor-Gene Q between PanPV and Zen8PV. Background fluorescence levels of the PanPV and updated Zen8PV assays were measured against a virus panel of 32 polioviruses wild type 1. Fluorescence at cycle six was selected as representative of background levels for each assay and sample.

**Fig 4:**
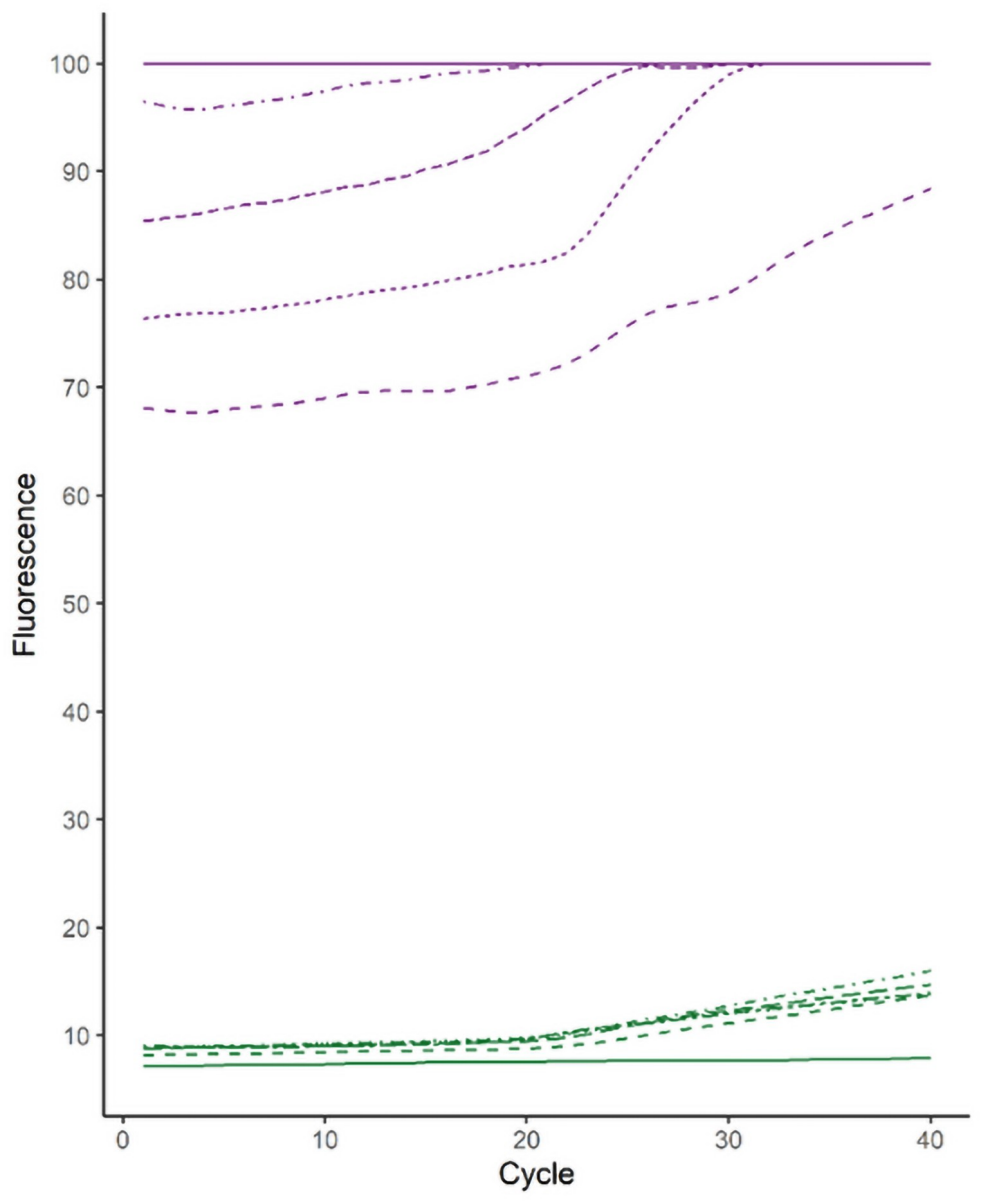
Comparative raw fluorescence data between PanPV and updated PanPV from six poliovirus isolates. Percentage of raw background fluorescence values from standard PanPV assay, purple broken lines (n = 4) and purple solid lines (n = 2) that overlap at the maximum detectable signal (where samples increased 100%). Green lines are the fluorescence measured from the updated PanPV probes tested with the same isolates in the Rotor-Gene Q.

A Zen^™^ quencher was added as a second, internal quencher in the PanPV probe at the 8^th^, 9^th^, or 10^th^ base from the 5’ reporter dye sequence, respectively (S1 Table). The best probe (Zen at position 8) had reduced background compared to the standard PanPV probe (7.05 ± 0.35 and 61.02 ± 3.74 respectively). The updated PanPV probe with Zen^™^ at position 8 was selected because it showed the lowest background combined with the highest fluorescent signal when tested with synthetic poliovirus RNA transcripts (Sabin 1, Sabin 2 and Sabin 3; Fig 2). A total of 184 virus isolates (including 32 WPV1 isolates that were of programmatic importance) were tested in parallel using both PanPV and updated PanPV assays (Table 1). The WPV1 isolates showed a lower average background fluorescence in the updated PanPV probe than the standard PanPV probe (8.13 ±0.001 and 68.01 ± 0.15, respectively); the difference was statistically significant (*P*< 0.05) (Fig 3). Six false-negative samples previously missed on the Rotor-Gene Q by the standard PanPV assay were positive with the updated PanPV probe (Fig 4). All poliovirus serotypes were detected by the updated PanPV assay when tested on the ABI7500, CFX96, MX3000P, and Rotor-Gene Q. Interpretation of results was simplified because the updated PanPV assay significantly reduced background signals. Even weaker positive signals were more defined, with curves clearly separated from the background and a higher signal-to-noise ratio. The interpretation remained the same for the other PCR platforms that were evaluated.

### Comparable limit of detection for updated PanPV and standard PanPV probes

The 95% LOD was determined with three plaque-purified polioviruses representing three wild type 1 poliovirus genotypes as part of the quality assessment for any new assay deployed to the GPLN to show non-inferiority. The updated Zen8PV assay maintained the same sensitivity as the PanPV assay: the LOD was 1 CCID_50_·μl^-1^ for Sabin 1 reference virus and 10 CCID_50_·μl^-1^ for WPV1-SOAS and AFRO-WEAF-B1 reference virus templates, the same LODs as previously identified (Table 3).

**Table 3:** Limit of detection for poliovirus serotype 1 with PanPV and updated PanPV assays tested with 20 replicates.

Reference Virus Strain*	Assay	No. of positive wells by virus titer (CCID50/μL)^#^				95% LOD (CCID50/μL)
		10³	10²	10¹	1	
PV1-Sabin	PanPV	n.d.	20	20	8	10¹
	Zen8PV	n.d.	20	20	12	10¹
WPV1-WEAF-B	PanPV	n.d.	20	6	0	10²
	Zen8PV	20	20	13	n.d.	10²
WPV1-SOAS	PanPV	20	19	9	n.d.	10²
	Zen8PV	20	20	9	n.d.	10²

*Assay was performed using the three-reference poliovirus strains: Sabin 1, accession no. AY184219; WPV1-WEAF-B, KY941931; WPV1-SOAS, KY941934.

^#^Pfu/μl: plaque-forming unit per microliter.

### PanPV probe with double quencher showed comparable results to PanPV with single quencher in five GPLN laboratories in pilot tests

The new PanPV assay was piloted in GPLN laboratories that have AFP surveillance samples of programmatic importance from three WHO regions. The National Institute of Biomedical Research (INRB, Democratic Republic of Congo); Institut Pasteur in Madagascar; the National Institute of Virology Mumbai (NIVMU, India); the Research Institute for Tropical Medicine (RITM, Philippines); and the National Institute of Health (NIH, Pakistan) pilot tested the Zen PV assay. In collaboration with these GPLN partners, the updated PanPV assay was validated screening 293 poliovirus and non-poliovirus isolates (n = 17) from AFP surveillance at the INRB (n = 18); Pasteur Institute (n = 50); NIVMU (n = 41); RITM (n = 75); and NIH (n = 126). Most of the virus isolates were serotyped as Sabin 1 or Sabin 3 (n = 263) [Table 4]. Both PanPV assays, using the newly designed Zen8PV probe and the standard PanPV probe, were run on the ABI7500 concurrently on the same plates for Ct value comparison.

**Table 4:** Number of poliovirus isolates by genotype (Sabin 1, Wild Poliovirus 1 etc.) pilot tested with Zen8PV and PanPV assays by five GPLN laboratories*.

Serotype	RITM-Philippines	NIH-Pakistan	Institut Pasteur- Madagascar	NIVMU—India	INRB-DRC	Total
Sabin 1	34	74	39	14	2	163
Sabin 3	31	33	7	26	3	100
Wild 1		17				17
Poliovirus 2					13	13
Negative^^^	10	2	4	1		17
Total	75	126	50	41	18	310

*RITM-Research Institute for Tropical Medicine, Philippines; NIH-National Institute of Health, Islamabad, Pakistan; NIVMU-National Institute of Virology, Mumbai, India; INRB-National Institute of Biomedical Research, The Democratic Republic of the Congo; Institut Pasteur, Madagascar.

^^^Negative for poliovirus after standard GPLN virus isolation.

**Fig 5:**
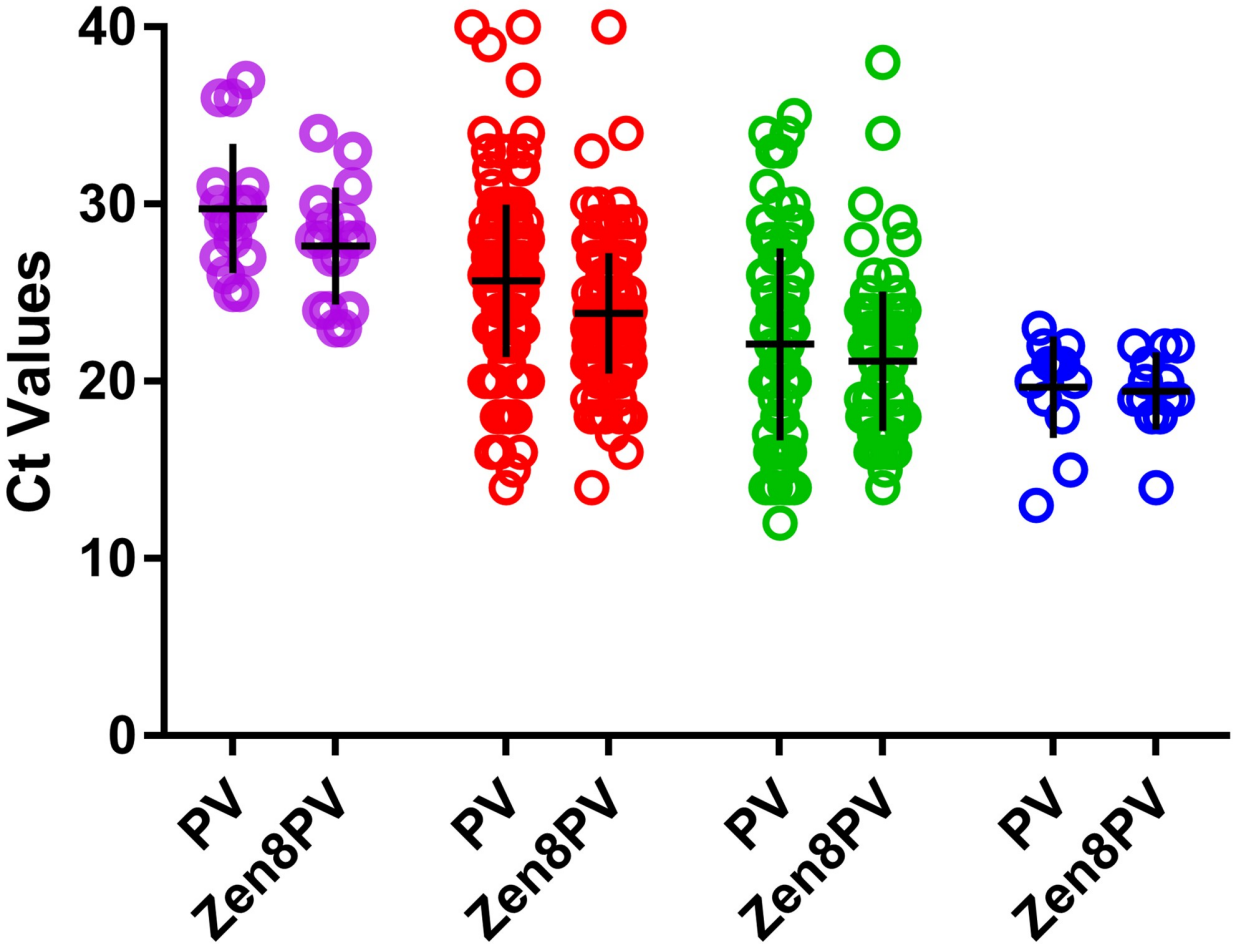
Pilot testing of poliovirus isolates with PanPV and updated PanPV probes. A total of 293 poliovirus isolates were run with both PanPV and Zen8PV probes. Ct values are shown for wild poliovirus type 1 (purple); Sabin 1 (red); Sabin 3 (green); poliovirus type 2 (blue). The cross depicts the mean Ct value with standard deviation of all positive isolates.

The specificity and sensitivity of the updated PanPV assay were 100% concordant in non-Rotor-Gene Q platforms compared to the previous version of the PanPV assay. In Rotor-Gene Q platforms, the updated PanPV assay did not have false negatives, unlike the previous version of the PanPV assay. The mean Ct values of the PanPV assay and Zen8PV were 24.3 and 22.9, respectively. The Zen8PV assay was 1–2 Ct’s lower compared to the standard PanPV assay (Fig 5), and interpretation of results was simplified due to reduced background with higher signal-to-noise ratios.

## Discussion

Rapid poliovirus detection from poliovirus isolates remains a crucial component of laboratory surveillance. We evaluated four machines, Rotor-Gene Q, Stratagene MX3000P, Bio-Rad CFX96, and the ABI7500, for performance with the GPLN assays. Previous versions of the ITD polio diagnostic real-time RT-PCR assays contained a standard PanPV assay (versions 1 through 5). We validated a new updated PanPV assay with Zen^™^ quencher to increase sensitivity and interpretation based on the reduced background noise for the most common real-time PCR platforms in the network. All real-time PCR platforms had concordant results with the appropriate run profiles. The updated PanPV probe improved poliovirus detection by reducing background signals and making overall analysis simpler in both the poliovirus panel and in the pilot study. The validation of various platforms and the release of an updated PanPV assay will increase the robustness of the assays used by the GPLN and will decrease time spent analyzing data.

Though the majority of its 114 polio diagnostic labs use ABI7500, the GPLN also includes laboratories using alternative real-time RT-PCR platforms, such as Rotor-Gene Q and Stratagene MX3000P. The rationale for choosing different platforms includes many reasons such as compatibility with other (non-polio) assays, institutional standardization, local sales and service, or other instrument availability. The advantage of real-time systems like the BioRad CFX96 and the Rotor-Gene is that they do not necessitate bi-annual calibrations. Since they use light emitting diodes (LED) as their light source, no lamp changes are needed, unlike the halogen lamps used in the AB7500 or Mx3000.

The high nucleotide sequence diversity among polioviruses presents a challenge to the design of nucleic acid-based assays. Genomic sequences that encode strong amino acid conservation can still be highly variable because of codon degeneracy. To accommodate this variability, degenerate codon positions on the template were matched by mixed-base or deoxyinosine residues on the primers and probe. The specificity of the updated PanPV assay was 100% (184 out of 184 poliovirus and non-poliovirus isolates) and the sensitivity was 1 to 100 CCID_50_·μl^-1^ (Sabin 2, and WPV1 respectively). The PanPV real-time RT-PCR assay in the ITD 5.0 has excellent diagnostic specificities for a diverse array of poliovirus genotypes (100%). However, this results in higher background signals, leading to the potential misinterpretation of results as false-negative.

To replace the old version of the PV assay, we updated the PanPV assay by adding a Zen^™^ quencher as a second internal quencher within the PanPV probe in the 8^th^ position from the 5’ end of the probe sequence. The updated PanPV assay sensitivity and specificity were assessed with 15 non-polio enteroviruses, 11 CPE-positive enterovirus-negative samples, and 158 virus isolates, including all PV serotypes and relevant genotypes circulating in the past decade. One limitation of this study was that the limited number of non-polio enterovirus isolates available due to the selective nature of the L20B cells used for virus isolation. Multi-site validation of the updated PanPV assay in the GPLN showed identical or better results compared to the previous PanPV assay. In addition, the new updated PanPV assay reduced background signals, which simplified interpretation of results.

A limitation of the updated PanPV pilot testing was the sample size: only 310 virus isolates were parallel tested with the updated and standard PanPV assay; most of which were Sabin 1 and Sabin 3 viruses, because there were not many wild PV isolates and only 17 PV type 2 to include in this comparison. This was primarily due to the advanced state of the global eradication program, where wild type 1 poliovirus is found in only Afghanistan and Pakistan in the WHO EMR region. In addition, the Rotor Gene Q cycler was used without an approved run method, thus a head-to-head comparison using the same cycler was not possible. The pilot testing was performed in laboratories that had both, programmatically important AFP surveillance samples (i.e. WPV1, PV2) and an approved cycler (i.e. AB7500). In August 2020 the WHO AFR region was certified wild poliovirus-free and it is now increasingly difficult to test assays prospectively. Retrospective testing is also becoming challenging since even potentially infectious material that might contain poliovirus type 2 has been discarded in order to comply with World Health Organization GAPIII requirements [15]. Since its development and piloting, the new PanPV assay has been deployed to the GPLN for use in 2019 to laboratories that use Rotor Gene Q cyclers among others.

The work described here serves one key purpose. It establishes a uniform standard for future ITD evaluations by providing a baseline to screen alternative platforms suited to a lab’s financial, scientific, diagnostic needs, as well as considerations of the GPLN. The continuous update and validation of methods and procedures are critical for any network, whether domestic or international, to incorporate new technologies and to improve detection sensitivity. Diagnostic networks must be prepared to handle evolving issues, both human-made, like logistics and rules and regulations, and molecular evolution of the pathogen (e.g. genetic drift). The example of the GPLN illustrates the need for collaboration and necessary background work required for a functional global network, which can serve as a model for future global laboratory networks.

## Supporting information

S1 Table Updated PanPV probe nucleotide sequences with the Zen-labeled nucleotide in boldface type. *Probes labeled with FAM quenched with Iowa Black Quencher, R = A or G; N = A/C/G/T; I = Inosine base analog pairs with A/C/G/T.
(DOCX)
