# Correction: Validation of a redesigned pan-poliovirus assay and real-time PCR platforms for the global poliovirus laboratory network

**DOI:** 10.1371/journal.pone.0308467

**Published:** 2024-08-02

**Authors:** Hong Sun, Chelsea Harrington, Nancy Gerloff, Mark Mandelbaum, Stacey Jeffries-Miles, Lea Necitas G. Apostol, Ma. Anne-Lesley D. Valencia, Shahzad Shaukat, Mehar Angez, Deepa K. Sharma, Uma P. Nalavade, Shailesh D. Pawar, Elisabeth Pukuta Simbu, Seta Andriamamonjy, Richter Razafindratsimandresy, Everardo Vega

There are errors in Table 2. The extension step information should have been 72°C/5 sec instead of 95°C/15 sec and the Reverse Transcription (RT)-Step information is not indicated. Please see the correct Table 2 here.

**Table 2:** Validated ITD 5.0 run conditions on different real time systems.

ABI7500 & 7500 fast			CFX96 & MX3000			Rotor-Gene Q		
RT‐Step	50°C	30min	RT‐Step	50°C	30min	RT‐Step	50°C	30min
	95°C	1min		95°C	1min		95°C	1min
PCR Cycles	95°C	15 sec	PCR Cycles	95°C	15 sec	PCR Cycles	95°C	15 sec
(40X)	50°C	45 sec	(40X)	50°C	45 sec	(40X)	50°C	45 sec
	25% ramp rate						61°C	20 sec
	72°C	5sec		72°C	5sec		72°C	5sec
