# Supplementary material for: Validation of a redesigned pan-poliovirus assay and real-time PCR platforms for the global poliovirus laboratory network
Source: PLoS One. 2021 Aug 6;16(8):e0255795. doi: 10.1371/journal.pone.0255795 (PMC8345876; doi:10.1371/journal.pone.0255795)
Supplement: S1 Table — *Probes labeled with FAM quenched with Iowa Black Quencher, R = A or G; N = A/C/G/T; I = Inosine base analog pairs with A/C/G/T. (DOCX) [file pone.0255795.s001.docx]

**S1 Table. Updated PanPV probe nucleotide sequences with the Zen-labeled nucleotide in boldface type.**

| Probe name | Oligonucleotide sequence (5' - 3’) * |
| --- | --- |
| Zen10PV | TGR TTN ARI **G**CR TGI CCR TTR TT |
| Zen9PV | TGR TTN AR**I** GCR TGI CCR TTR TT |
| Zen8PV | TGR TTN A**R**I GCR TGI CCR TTR TT |
